# RD-YOLO: An Effective and Efficient Object Detector for Roadside Perception System

**DOI:** 10.3390/s22218097

**Published:** 2022-10-22

**Authors:** Lei Huang, Wenzhun Huang

**Affiliations:** School of Electronic Information, Xijing University, Xi’an 710123, China

**Keywords:** feature extraction, feature fusion, attention mechanism, roadside images, object detection, YOLOv5s

## Abstract

In recent years, intelligent driving technology based on vehicle–road cooperation has gradually become a research hotspot in the field of intelligent transportation. There are many studies regarding vehicle perception, but fewer studies regarding roadside perception. As sensors are installed at different heights, the roadside object scale varies violently, which burdens the optimization of networks. Moreover, there is a large amount of overlapping and occlusion in complex road environments, which leads to a great challenge of object distinction. To solve the two problems raised above, we propose RD-YOLO. Based on YOLOv5s, we reconstructed the feature fusion layer to increase effective feature extraction and improve the detection capability of small targets. Then, we replaced the original pyramid network with a generalized feature pyramid network (GFPN) to improve the adaptability of the network to different scale features. We also integrated a coordinate attention (CA) mechanism to find attention regions in scenarios with dense objects. Finally, we replaced the original Loss with Focal-EIOU Loss to improve the speed of the bounding box regression and the positioning accuracy of the anchor box. Compared to the YOLOv5s, the RD-YOLO improves the mean average precision (mAP) by 5.5% on the Rope3D dataset and 2.9% on the UA-DETRAC dataset. Meanwhile, by modifying the feature fusion layer, the weight of RD-YOLO is decreased by 55.9% while the detection speed is almost unchanged. Nevertheless, the proposed algorithm is capable of real-time detection at faster than 71.9 frames/s (FPS) and achieves higher accuracy than the previous approaches with a similar FPS.

## 1. Introduction

Road traffic accidents are considered the most important general health concern, as it results in numerous injuries and deaths worldwide. The survey indicated that human error is the main cause of most vehicular accidents [1]. Drivers need to be highly focused and always pay attention to changes in the surrounding environment while driving a conventional car. However, the judgment of drivers can be affected by many factors such as fatigue, noise, and weather, which contribute to the risk of road traffic accidents. With the arrival of the information age, Cooperative Intelligent Transportation Systems (C-ITS) [2] have emerged. The vehicle–road cooperation system mainly equips vehicles and roadsides with advanced sensors to sense the road traffic environment in real time. With the availability of cooperative information regarding vehicles and road conditions, human error can be effectively avoided. Hence, vehicle–road cooperation is attracting increasing attention over the past few years.

The vehicle–road cooperative system contains two parts, i.e., the perception part and the cooperation part. The perception part is primarily roadside perception; vehicle perception is supplementary. The cooperation part uses Cooperative Vehicle-to-Everything (C-V2X) communication technology to realize the cooperative and integrated sensing of road. The intelligent perception system is an important antecedent of autonomous driving [3]. Vehicle perception mainly equips autonomous vehicles with sensors to achieve automatic decisions by analyzing recently acquired road and obstacle information in space [4]. However, there are obvious shortcomings in relying on vehicle perception alone [5]. Firstly, vehicle sensors are unable to achieve the multilevel and long-distance perception of the environment with perceptual blind spots, which makes it difficult to obtain effective traffic information at long distances. Secondly, perception vehicles need to install multiple sensors (e.g., LiDAR, millimeter-wave radar, high-definition cameras, among others) to obtain more comprehensive environmental information, thus requiring a more complex and expensive system. Finally, for a long time in the future, intelligent and nonintelligent vehicles will coexist, while the penetration rate of perception systems for intelligent vehicles is relatively low. To make up for the lack of vehicle perception, combining roadside perception to sense road conditions is an efficient approach. Therefore, it is necessary to study the roadside perception technology of the vehicle–road cooperative system.

The main purpose of roadside perception is to improve the over-the-horizon perception capability of intelligent vehicles [6], which can expand the perception range of intelligent networked vehicles and make early warning alerts. As the main method of roadside perception, visual target detection is mainly aimed at identifying traffic objects on the road, which can visualize the current road conditions. However, the roadside images obtained by visual sensors contain a large number of small-scale targets due to the wide roadside view. The traditional target detection model extracts less information about the shallow features [7,8], which makes it difficult to accurately classify and precisely localize the small-scale targets. Especially in complex road conditions, the overlap and occlusion of the dense objects are more obvious, which leads to a higher rate of missed detection and misdetection. Moreover, the roadside object scale varies violently due to the different heights of the visual sensors installed. Finally, the information sensed by the roadside perception system needs to be transmitted to the vehicle for decision and control via wireless communication technology [9], which has high requirements for real-time and efficient deployment of detection algorithms. Hence, designing an effective and efficient algorithm for roadside object detection is a pressing problem.

With the rapid development of deep learning in machine vision, the target detection algorithms based on deep learning are mainly classified into two categories. One is the two-stage object detection algorithm represented by the SPP-Net [10] and R-CNN series algorithms [11,12,13], which cannot meet real-time requirements in terms of detection speed due to structural limitations. Another is the one-stage object detection algorithm represented by the SSD [14] and YOLO series algorithms [15,16,17]; by performing classification and regression tasks at the same time as generating boxes, the detection speed is significantly improved. Currently, a lot of research has been carried out in the field of road traffic vision. To analyze the movement behavior of traffic objects, Murugan et al. [18] used two-stage object detection algorithm R-CNN for vehicle identification in the traffic monitoring system. Due to the poor performance of RCNN algorithm for small-target detection, Liang et al. [19] proposed a sparse R-CNN combining coordinate attention mechanism with ResNeSt to obtain better performance for traffic sign detection by edge devices equipped with self-driving vehicles. The R-CNN series algorithm has some advantages in object detection accuracy, but the detection speed is lower than YOLO. The YOLO series algorithm uses the idea of regression, which makes the generalized algorithm easier to learn and solves the problem of target characteristics and speed. For poor detection performance of small targets in autonomous driving, Benjumea et al. [20] optimized the feature extraction ability of YOLOv5 for small targets by redirecting the feature maps delivered to the Neck network and head layer. Du et al. [21] used YOLOv5 for pavement defect detection and introduced the BiFPN structure and Varifocal Loss in the YOLOv5 algorithm, which effectively improved the performance of pavement defect detection. 

As aforementioned, many methods achieve better target detection performance in the field of intelligent transportation, such as traffic sign detection, vehicle detection, and pavement defect detection. However, these models are difficult to deploy on roadside edge devices due to the increasing complexity of the model network. To solve the problems in the existing models, this paper investigates the current, more lightweight and flexible detector, YOLOv5. According to the characteristics of target detection in the roadside view, this paper optimizes YOLOv5 and proposes the roadside object detection algorithm RD-YOLO. To implement the real-time target recognition and efficient deployment of an object detection algorithm for roadside perception systems, the following work has been carried out in the paper:

(1) Based on the unique characteristics of roadside images, this study proposes RD-YOLO roadside object detection algorithm by optimizing the network, channels, and parameters. Compared with the latest roadside target detection algorithms, the proposed model has qualitative improvements in speed and accuracy, while the model volume is significantly reduced to facilitate the deployment of edge devices.
(2) Aimed at the problem of high complexity and poor performance of small-scale target detection in the current algorithms, we reconstructed the feature fusion layer by adding the 32× downsampling feature fusion layer and removing the 4× downsampling feature fusion layer, which improves small-scale object detection accuracy and compresses the volume of the model.
(3) We replaced the original pyramid network with GFPN to deal with large-scale variance of objects, which improves the aggregation capability of multiscale features and the adaptability of the network to different scale features.
(4) To solve the problems of poor performance for small-target detection in object-dense scenarios, we integrated the CA attention mechanism into the YOLOv5s Backbone network to enhance the important channel and spatial feature information in the features, which can accurately localize and identify important information.
(5) For slow convergence and inaccurate regression results in the small-target detection, we improved the loss function of the YOLOv5s prediction head to accelerate the learning of high confidence target, which effectively improves the speed of the bounding box regression and the positioning accuracy of the anchor box.

## 2. Related Work

This section describes the limitations of roadside object detection in detail. By analyzing the existing object detection algorithms, YOLOv5 is chosen as the benchmark model. Finally, the YOLOv5 network structure is introduced and its current problems are analyzed.

### 2.1. Roadside Object Detection

**Figure 1:**
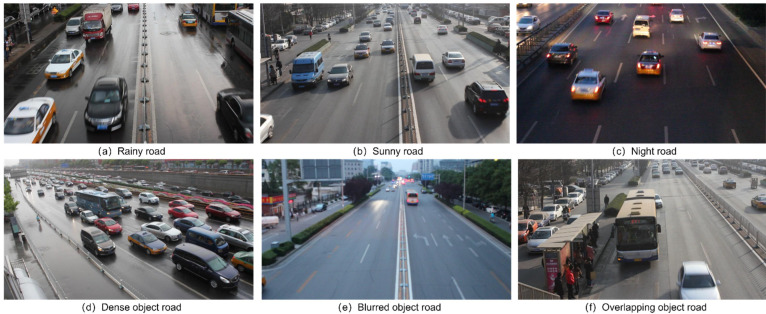
Complex actual roadside images.

The roadside cameras are installed on different road environments, including day, night, overlapping, obscured, dense, etc. As shown in Figure 1, roadside object detection exposes three problems. First, the object scale varies violently because of the different installation heights of the roadside cameras. Second, in rainy and night road environments, the captured images tend to contain more blurred targets; especially in complex traffic road conditions, the overlap and occlusion of the dense objects are more obvious. Third, the roadside images contain a large number of small-scale targets that are not easily identified because of the wide roadside view. The above three problems make roadside target detection very challenging.

Currently, the main object detection algorithms are based on convolutional neural networks for improvement. Although the convolutional neural network can effectively extract feature information, the locality of convolutional operation limits its ability to obtain global context information. Some scholars have explored new methods. For multitarget detection in traffic scenes, Wang et al. [22] proposed a novel detection framework, AP-SSD, by introducing a feature extraction convolutional kernel library, which enables the network to select convolution kernels adaptively. Although it improves detection accuracy, it also brings an increase in computation. Zhu et al. [23] proposed a multisensor multilevel enhanced convolutional network structure (MME-YOLO) based on YOLOv3, which effectively improved the average detection accuracy value on the UA-DETRAC dataset by adding cross-level attention blocks and an image composite module. To improve the target detection capability and efficiency of the detector for autonomous driving, Cai et al. [24] introduced the CSPDarknet53 structure and five scale detection layers in the YOLOv4 algorithm to improve the detection accuracy, and the lightweight modification of the model by network pruning effectively improved the inference speed. To solve the problems of misdetection and missed detection of small targets in complex traffic scenes, Li et al. [25] proposed a method that combines depth information obtained by the end-to-end PSMNet with the YOLOv5s target detection algorithm to improve the feature extraction ability of small targets, which improves the detection accuracy of small-scale targets.

Although the emergence of large neural networks improves the performance of detection, it is followed by the problem of efficiency. Because roadside object detection algorithms need to be deployed on edge computing devices, complex network models cannot be satisfied for use in roadside perception systems with relatively poor computational power. Therefore, this paper proposes an improved object detection model, RD-YOLO, to solve the problem of the speed and accuracy of roadside object detection being unable to be improved at the same time due to small targets, complex backgrounds, and limited feature extraction. 

### 2.2. YOLOv5 Network Structure

**Figure 2:**
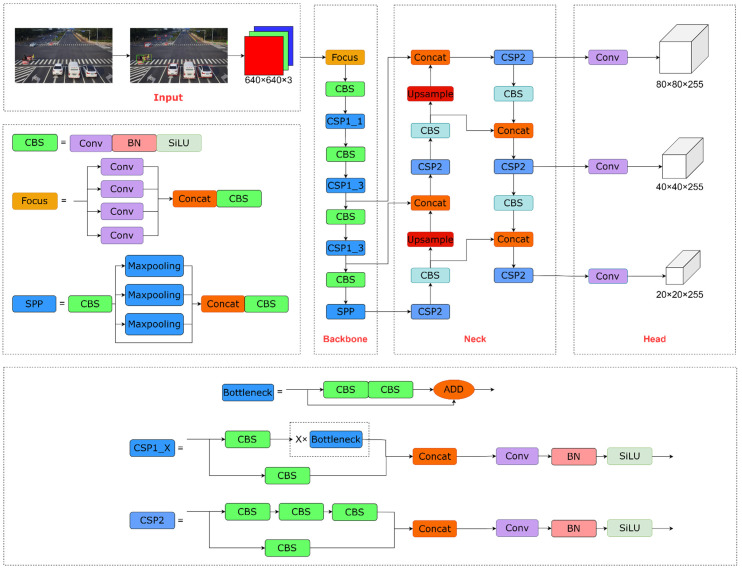
YOLOv5s network structure.

The YOLO series algorithm is a target detection algorithm based on deep learning and the convolutional neural network, which has the advantages of fast inference, high detection accuracy, and real-time detection. With the rapid development of deep learning in machine vision, the YOLOv5 algorithm has emerged. Compared to previous generations of algorithms, YOLOv5 has higher accuracy, faster speed, and smaller size. YOLOv5 contains four derived models, including YOLOv5s, YOLOv5m, YOLOv5l, and YOLOv5x. They have the same model architecture, but the model depth and width increase sequentially. Therefore, YOLOv5s was chosen as the benchmark model to build the roadside object detection algorithm. The YOLOv5s network structure is shown in Figure 2.

The YOLOv5s framework consists of four parts, including the Input layer, Backbone network, Neck network, and Head layer. The main purpose of the Input layer is to perform preprocessing operations on the input images. The Input layer contains mosaic data enhancement, adaptive anchor box calculation, and adaptive image scaling. They effectively improve the efficiency of extracting features from the input images. YOLOv5 employs the Focus module, CSP structure [26], and spatial pyramidal pooling (SPP) module [27,28] as the Backbone, which solves the problem of the detection algorithm requiring a large number of calculations during the push process. The Neck network consists of a feature pyramid network (FPN) and path aggregation network (PANet); the high-level feature and the output of different layers of the CSP module are aggregated by the top-down pathway, and then the shallow features are aggregated by the bottom-up pathway, which fully integrates the image feature of different layers. As the final detection, the Head layer contains the bounding box loss function and non-maximum suppression (NMS) [29]. The output of the Neck network is input to the Head layer to generate prediction boxes, and then the prediction boxes with local regional redundancy are filtered out to obtain the final detection results by NMS operation.

### 2.3. Problems of YOLOv5 Algorithm

As a one-stage object detection algorithm, YOLOv5 has a significant improvement in detection speed compared to two-stage object detection algorithms such as Faster R-CNN and Mask R-CNN [30], which meeting the requirement of real-time detection. However, in terms of detection accuracy and model weight, it still needs to be improved in practical production with complex backgrounds.

Although YOLOv5s in the YOLOv5-derived algorithms greatly simplifies the complexity of the network structure by reducing the depth and width, it also reduces the detection accuracy. YOLOv5s contains multiple convolutional modules, so the size of the feature map decreases as the number of downsampling convolution layers increases during feature information extraction. Therefore, it is difficult for YOLOv5s to accurately classify and precisely localize the small-scale targets due to the small number of shallow features extracted, which leads to the problem of misdetection and missed detection in small-scale target detection.

The YOLOv5 Neck network with FPN + PANet combined structure focuses on the deep feature fusion, which weakens small target detection and indirectly increases interference noise by upsampling operation. However, in roadside object detection, the timely detection of small or distant targets is important for safe driving. Therefore, this paper improves the model and further enhances its roadside object detection capability.

## 3. Proposed Method

This section describes in detail the small-target feature extraction method, the method of feature fusion, the attention mechanism, and the improvement of the loss function. Finally, a lightweight and accurate roadside object detection method based on YOLOv5 is proposed.

### 3.1. Feature Fusion Layer Reconstruction

The roadside target detection algorithm not only needs to accurately identify targets in complex road environments but also needs to compress the size of the model as much as possible for deployment in roadside edge devices. Moreover, the field of perception is broader in the roadside view; the roadside images contain a large number of small-scale targets. Therefore, the Backbone network and Neck network of the YOLOv5s model are modified in this study. Under the premise of ensuring detection performance, the network parameters and model calculations are reduced in order to realize the lightweight and improved design of the target detection network.

**Figure 3:**
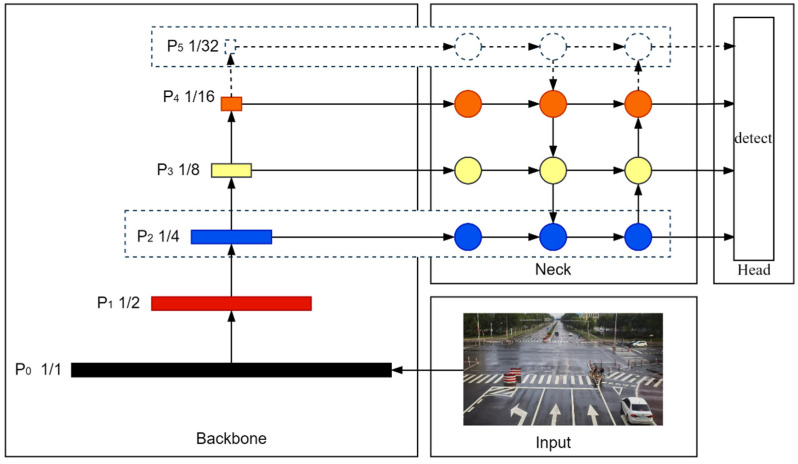
The network architecture of the improved feature fusion layer. The 32× downsampling feature fusion layer is removed. The 4× downsampling feature fusion layer is added.

The Backbone network contains four downsampling modules, which extract features at different levels in the image by deep convolution operation. However, in the Backbone network, the small-target features decrease or even disappear with the increase in feature levels due to the multiple uses of downsampling. Therefore, the improved design of the feature fusion layer is executed in this study. The top feature extraction layer of the YOLOv5s Backbone network is removed, which reduces the complexity of the network and decreases the invalid information into the next stage at the same time. On the other hand, the low-level features contain more location and detailed information due to the small feature-space-receptive fields, which can accurately detect the small targets in roadside images. Therefore, the 4x downsampling feature fusion layer of the YOLOv5s Neck network is added, which captures more effective information about small targets and improves the detection capability of small targets. The network architecture of the improved feature fusion layer is shown in Figure 3.

Compared with the original network architecture, the improved structure is more suitable for the detection of small targets in roadside images, which reduces the network complexity and improves the detection accuracy of the algorithm at the same time.

### 3.2. Multiscale Feature Fusion

Due to the different road conditions and the different heights of visual sensor installation, the target scale varies violently, which brings a great challenge to object recognition in roadside images. Especially in the case of complex road conditions, the target information is more complex and overlaps severely. In the feature extraction network, by continuously downsampling convolution layers, the extracted multiscale features are input into the Neck network for feature fusion. The low-level feature maps have a smaller feature-space-receptive field, which is more suitable for perceiving location and detailed information. However, due to the small number of downsampling feature extraction, the low-level feature maps have relatively less semantic information and contain more noise. The high-level features have more semantic information, but the feature-space-receptive field becomes large due to multiple convolution operations, which leads to poor perception ability of details in the image. Therefore, effective feature fusion of the extracted feature information is the key to improving the model detection performance.

**Figure 4:**
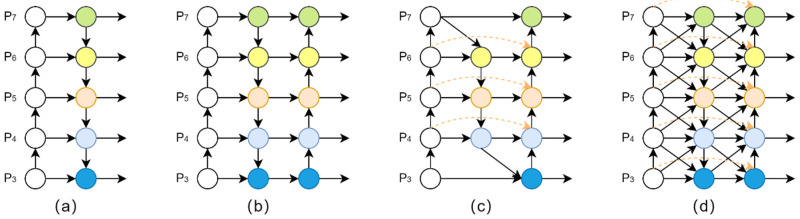
Iterative design of feature pyramid network from level 3 to level 7 (P3-P7). (**a**) FPN proposes a top-down pathway to aggregate multiscale features; (**b**) PANet adds an additional bottom-up pathway based on FPN; (**c**) BiFPN adds a bidirectional cross-scale pathway based on PANet; (**d**) GFPN introduces a queen-fusion pathway and skip-layer connection method based on PANet.

Feature fusion is currently the main method to deal with the multiscale discrepancy problem, and the representative algorithms are the feature pyramid network (FPN) [31], path aggregation network (PANet) [32], and bidirectional feature pyramid network (BiFPN) [33]. Their core idea is to aggregate multiscale features that is extracted from the Backbone network. However, these feature pyramid network structures mainly focus only on the scale of features but ignore the level of features. When the size of the detecting object is basically the same, it is difficult for the network to distinguish between objects with simple appearance and objects with complex appearance, because the feature map contains only single-level or few-level features. Therefore, a novel feature fusion method, the generalized feature pyramid network (GFPN), was proposed by Jiang et al. [34]. GFPN proposes a new cross-scale fusion that aggregates the feature of the same level and neighbor level, which provides more efficient information transfer. On the other hand, GFPN proposes a new skip connection method, which effectively prevents gradient vanishing in a heavy neck and expands into a deeper network. Under different floating-point operations per second (FLOPs) performance balances, GFPN has more excellent performance than other SOTA solutions, and the network structure is shown in Figure 4d.

**Figure 5:**
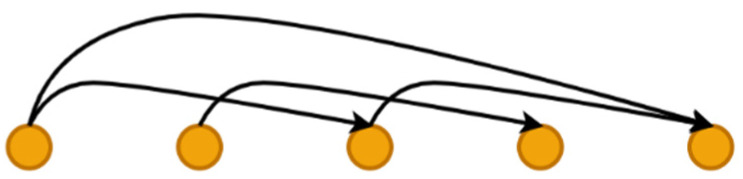
$log_{2}n-link$ skip-layer connection.

Since the GFPN structure has a higher complexity compared to other feature pyramid network structures. To avert the vanishing gradient problem during the increase in network computational volume, a new skip-layer connection method was proposed and named $log_{2}n-link$, which not only improves the expansion depth of GFPN but also preserves effective features for reuse, as shown in Figure 5.

**Figure 6:**
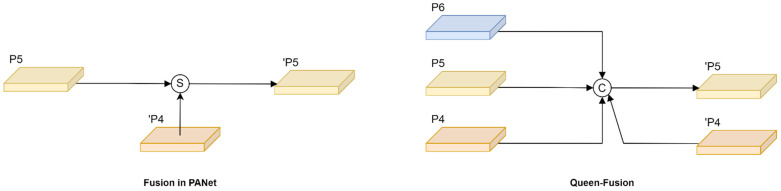
The improved design of cross-scale connection structure. S and C refer to the summation and concatenation, respectively. ‘P_k_ refers to the node at the next layer.

Sufficient information exchange should contain both skip-level connection and cross-scale connection. However, previous works in aggregating features of adjacent layers only consider the same-level feature or previous-level feature, which leads to poor performance in scenarios with large-scale variance of objects. Therefore, queen fusion was proposed to overcome large-scale variation, and the structure is shown in Figure 6. Each node receives input not only from the previous node but also from the nodes above and below it diagonally, which helps target features for effective information transfer and improves the adaptability of the network to different scale features. Moreover, the fusion style of GFPN uses the concatenation method instead of the summation method, which effectively reduces the loss of feature fusion.

### 3.3. Attention Mechanism

The YOLOv5 neck network structure focuses on the fusion of deep features, which weakens the detection of small targets. Especially in the scenario with dense objects, the detection accuracy of small targets is low. Moreover, due to multiple downsampling operations, the receptive field of the high-level feature map is relatively large, and a large amount of detailed information has been lost; small-target features are especially likely to be completely missing. To reduce the loss of small-target features during feature extraction and improve the ability of small-target detection, we introduce an attention mechanism that constructs a hierarchical attention structure similar to human perception to enhance the network feature extraction capability.

**Figure 7:**
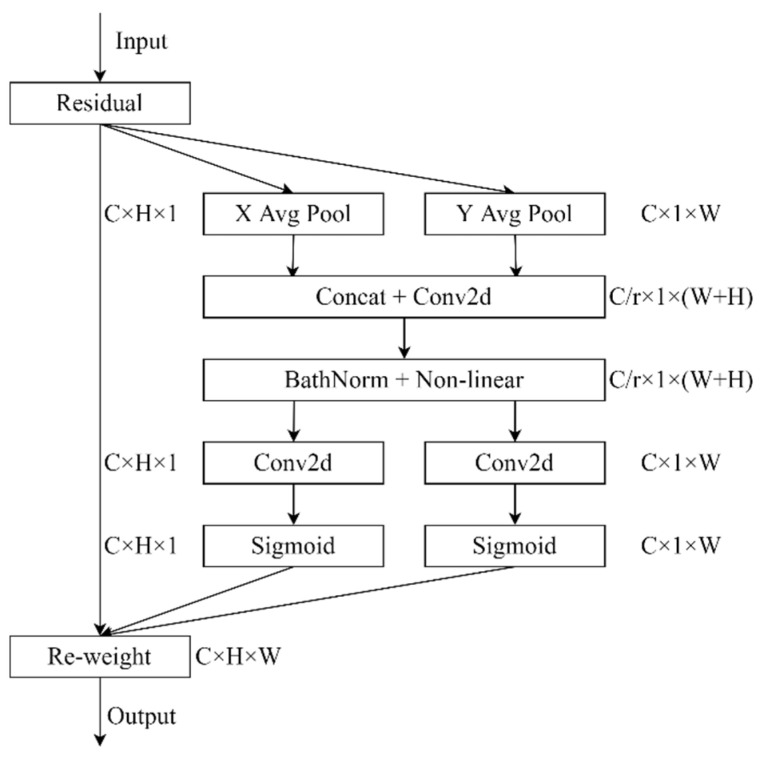
The CA attention mechanism, ‘X Avg Pool’ and ‘Y Avg Pool’ denote 1D horizontal global average pooling and 1D vertical global average pooling.

Coordinate attention (CA) is a network structure proposed by Hou et al. [35]; the main idea is to embed location information into channel attention. Channel relationships and long-range dependencies with precise positional information are more beneficial for the network to extract important information from feature images. The CA attention mechanism consists of two main components, i.e., coordinate information embedding and coordinate attention generation. As shown in Figure 7, given the input X, two spatial extensions of pooling kernels (1×W) and (H×1) are used to encode each channel along the horizontal coordinate and the vertical coordinate, respectively. The outputs $z^{w}$ and $z^{h}$ are concatenated and then sent to a shared (1×1) convolutional transformation. The concatenated feature maps are sent to BatchNorm and Nonlinear to encode the spatial information in the vertical and horizontal directions. The output $f\epsilon R^{C/r\times 1\times \left(W+H\right)}$ is split along the spatial dimension into two separate tensors, $f^{w}\epsilon R^{C/r\times 1\times W}$ and $f^{h}\epsilon R^{C/r\times H\times 1}$. Another two (1×1) convolutional transformations are utilized to separately transform $f^{w}$ and $f^{h}$ to tensors with the same channel number to the input X, yielding $f\epsilon R^{C\times 1\times W}$ and $f\epsilon R^{C\times H\times 1}$. Then, the attention weight maps $g^{h}$ and $g^{w}$ in two spatial directions are obtained after the activation function Sigmoidx [27], each attention weight feature maps carries long-range dependencies along a particular direction. Finally, the input feature map is multiplied with two weights, which enhances the expressiveness of the feature map.

**Figure 8:**
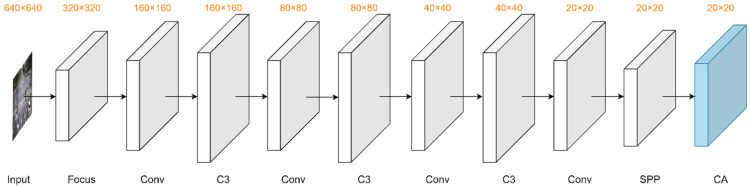
The Backbone structure with the CA module. We integrate the CA to enhance the correlation between feature channels. Then, the network can focus on important features in the roadside images.

In order to accurately identify and localize small targets in object-dense scenarios, we integrated the CA attention mechanism in the YOLOv5s Backbone network to enhance the important channel and spatial feature information in the features, and the improved Backbone network is shown in Figure 8. The CA attention mechanism added at the end of the Backbone network not only does not increase the network parameters and model computation but also facilitates the extraction of important feature information.

### 3.4. Loss Function

The loss function is used to measure the degree of overlap between the prediction boxes and the true boxes. For the slow convergence and inaccurate regression results in roadside small target detection, we introduce the Focal-EIOU Loss [36] which is more suitable for the regression mechanism. The original YOLOv5s model uses CIOU Loss [37] as the IOU loss function. The principle of CIOU Loss is as follows:

(1) $$L_{CIOU}=1-IOU+\frac{\rho^{2}(b,b^{gt})}{c^{2}}+\alpha v$$

(2) $$v=\frac{4}{\pi^{2}}{\left(\arctan\frac{w^{gt}}{h^{gt}}-\arctan\frac{w}{h}\right)}^{2}$$

EIOU Loss consists of three parts, i.e., the IOU loss, the distance loss, and the aspect loss. In this way, the first two parts of EIOU Loss continue the approach in CIOU Loss, which retains the profile characteristics of the CIOU Loss. Meanwhile, the aspect Loss directly minimizes the difference between target box’s and anchor box’s width and height, which effectively improves the converge speed and positioning accuracy. The principle of EIOU Loss is as follows:

(3) $$IOU=\left|\frac{A\cap B}{A\cup B}\right|$$

(4) $$\begin{array}{c} L_{EIOU}=L_{IOU}+L_{dis}+L_{asp}=\\ 1-IOU+\frac{\rho^{2}(b,b^{gt})}{c^{2}}+\frac{\rho^{2}(w,w^{gt})}{C_{w}^{2}}+\frac{\rho^{2}(h,h^{gt})}{C_{h}^{2}} \end{array}$$

There is a problem of imbalanced training examples in regression for bounding boxes, i.e., the number of high-quality anchor boxes with small regression errors in an image is much fewer than the number of low-quality examples with large errors, and the low-quality examples will produce too large a gradient to affect the training process. Therefore, Focal Loss is introduced to optimize the training examples imbalanced in the bounding box regression task, which separates high-quality anchor boxes from low-quality anchor boxes so that the regression process focuses on high-quality anchor boxes. The principle of Focal-EIOU Loss is as follows:

(5) $$L_{Focal-EIOU}=IOU^{\gamma }L_{EIOU}$$

where γ is a parameter that controls the degree of outlier suppression.

### 3.5. RD-YOLO Network Structure

**Figure 9:**
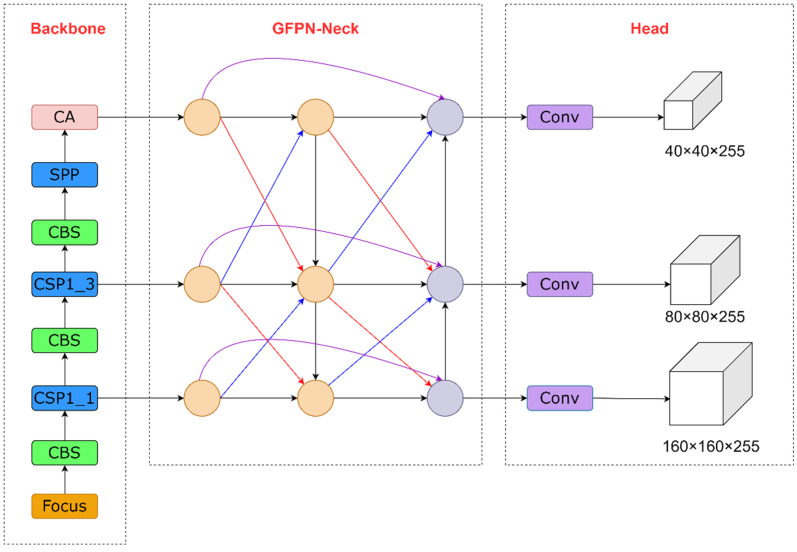
The improved network structure for RD-YOLO. The 32× downsampling feature fusion layer is removed. The 4× downsampling feature fusion layer is added. The CA attention mechanism is inserted behind the Backbone. The PANet is replaced by GFPN to fuse the multiscale features. The CIOU Loss function is replaced by Focal-EIOU Loss to enhance the regression efficiency.

According to the above improvement method, this paper proposes an effective and efficient object detector for the roadside perception system. The network structure of the improved algorithm is shown in Figure 9. 

To realize the lightweight deployment of the roadside target detection model and improve the detection accuracy of small targets, RD-YOLO removes the 32× downsampling feature fusion layer and adds the 4× downsampling feature fusion layer, which maximally preserves the feature information and improves the detection capability of small targets. The CA attention mechanism is introduced to the end of the Backbone network, which enhances the important channel and spatial feature information in the features and improves the ability to locate small targets. After that, the different resolution features that are extracted from the Backbone network are input into the Neck network for feature fusion. It contains both top-down, bottom-up, and queen-fusion information transfer paths. In the first path, the semantic information of deep features is passed downward to shallow features to enhance the multiscale semantic representation. In the second path, the detailed information of shallow features is passed upward to deep features to enhance multiscale localization. The aggregated feature maps contain both abstract semantic information and rich detail information, which effectively improves the positioning accuracy and classification precision of the target detection algorithm. In the final path, to aggregate more feature information at different levels, the nodes accept input not only from the previous level node but also from the nodes above and below it diagonally. Moreover, the nodes of the same layer are also connected to the output nodes to fuse more feature information, which helps target features for effective information transfer and improves the adaptability of the network to different scale features. Finally, the output of the GFPN network is input to the Head layer to generate prediction boxes, and then the prediction boxes with local regional redundancy are filtered out to obtain the final detection results by NMS operation.

## 4. Experiment

### 4.1. Dataset

**Figure 10:**
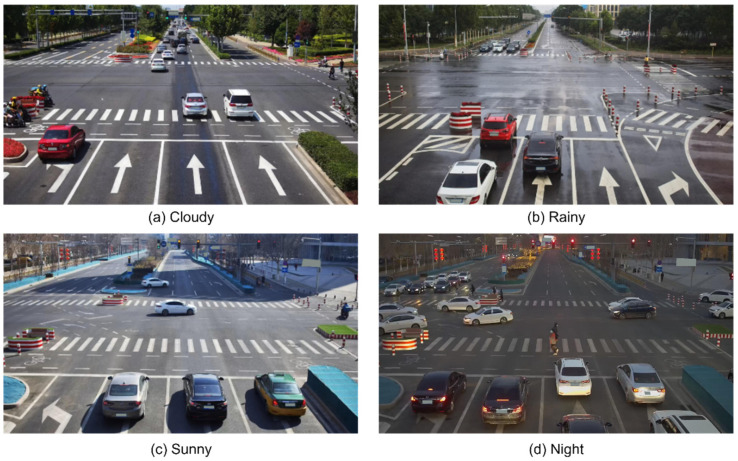
Some samples of the Rope3D dataset were collected under different scenarios and weather conditions.

In this paper, the Rope3D [38] dataset is used for the training and validation of the roadside object detection algorithm. Meanwhile, the annotation file is modified by using 2D boxes to label the detection targets. The Rope3D dataset is challenging. The roadside cameras are mounted on a roadside pole rather than on top of the car, so there are cases of different configuration conditions, including different parameters of the image acquisition equipment, different detection angles, and different installation heights. Moreover, the roadside view has a larger perception range, allowing more objects to be observed, which increases the difficulty of detection. Therefore, the Rope3D dataset fits well with the research context of this paper, and multiclass detection targets and multiple scenarios of the Rope3D dataset are beneficial to validate the performance of the model in various situations. Part of the roadside images is shown in Figure 10.

The Rope3D dataset contains 50 k images and over 1.5 M objects in various scenarios, which are collected under different road environments, including various lighting conditions (e.g., day, night, and dusk), different weather conditions (e.g., rainy, sunny, and cloudy), and different road scenes. To facilitate comparative experiment analysis, the Rope3D dataset is divided into different subdatasets based on scenarios, including cloudy, night, sunny, and rainy, as shown in Table 1.

### 4.2. Experimental Environment and Evaluation Metrics

#### 4.2.1. Experimental Environment

In this paper, the experiment was conducted using Windows 10 operating system with an Intel(R) Xeon(R) Silver 4210 CPU and an NVIDIA TITAN RTX GPU. The methods were implemented in Python 3.8 and Pytorch 1.6 was used as the deep learning framework. To ensure the accuracy of the training results, the algorithms involved in the comparison were tested under the same training parameters. The model training parameters were set as follows: the batch size was 8, the learning rate was 0.01, the momentum was 0.937, and the weight decay was 0.0005.

#### 4.2.2. Evaluation Metrics

In order to more accurately analyze the performance of the improved YOLOv5s object detection algorithm, it is critical to use appropriate evaluation metrics. In this paper, frames per second (FPS) and mean average precision (mAP) are employed as the evaluation metrics of this experimental algorithm model. Before introducing these metrics, the following concepts are introduced: true positives (TP) indicate the number of samples that are actually positive cases and classified as positive cases; false positives (FP) indicate the number of samples that are actually negative cases but classified as positive cases; false negatives (FN) indicate the number of samples that are actually positive cases but classified as negative cases. Then, we need to obtain the precision (P) and recall (R). The precision is the proportion of all targets predicted by the model that are correctly predicted. The recall is the proportion of all positive targets that are correctly predicted. The calculation formulas of precision and recall are as follows:

(6) $$P=\frac{\mathrm{TP}}{\mathrm{TP}+\mathrm{FP}}$$

(7) $$R=\frac{\mathrm{TP}}{\mathrm{TP}+\mathrm{FN}}$$

Average precision (AP) is the average of the precision of a class in the dataset. The precision–recall (P-R) curve was constructed by using recall as the horizontal axis and precision as the vertical axis, i.e., the P-R curve around the size of the area reflects the comprehensive performance of the model. The mean average precision (mAP) is the average of all classes of AP. The specific principle is as follows:

(8) $$\mathrm{AP}=\int_{0}^{1}P\left(R\right)d\left(R\right)$$

(9) $$\mathrm{mAP}=\frac{1}{N}\sum_{i=1}^{N}{\mathrm{AP}}_{i}$$

where *N* is the number of detected categories.

### 4.3. Experimental Result and Analysis

#### 4.3.1. Performance Comparison Analysis of Individual Structure Change

In order to verify the effect of individual structure changes on network performance. We proposed three algorithms to compare with the original YOLOv5s algorithm, as follows:

- YOLOv5s-T: reconstructing the feature fusion layer of YOLOv5s by removing the 32× downsampling feature fusion layer and adding the 4× downsampling feature fusion layer;
- YOLOv5s-G: transforming the feature pyramid structure of YOLOv5s by introducing the GFPN structure;
- YOLOv5s-CA: transforming the Backbone network by introducing the CA attention mechanism.

YOLOv5s, YOLOv5s-T, YOLOv5s-G, and YOLOv5s-CA were trained and tested, respectively, and the experimental results of the four algorithms are shown in Table 2.

Analysis of the experimental results shows the performance of the original YOLOv5s model on the dataset with a mAP@0.50 of 53.6%, FPS of 71.4, and weight of 13.83 MB. Compared to YOLOv5s, the YOLOv5s-T increased by 3.5% in mAP@0.50, increased by 14.8 in FPS, and decreased by 71.1% in weight, indicating that the structure was proposed to more effectively capture small target features and to compress the size of the model. The YOLOv5s-G replaces the original pyramid network with GFPN. Although the improved feature fusion structure brings an increase in computation, it significantly enhances the object detection accuracy of the algorithm. mAP@0.50 increased by 3%. Finally, the Backbone network is optimized by introducing the CA module, which improves the detection ability of dense small targets. The new Backbone network structure basically does not increase network parameters and model computations, resulting in an increase of 0.5% for mAP@0.50, which illustrates the effectiveness of the attention mechanism.

#### 4.3.2. Performance Comparison Analysis of Loss Function

**Figure 11:**
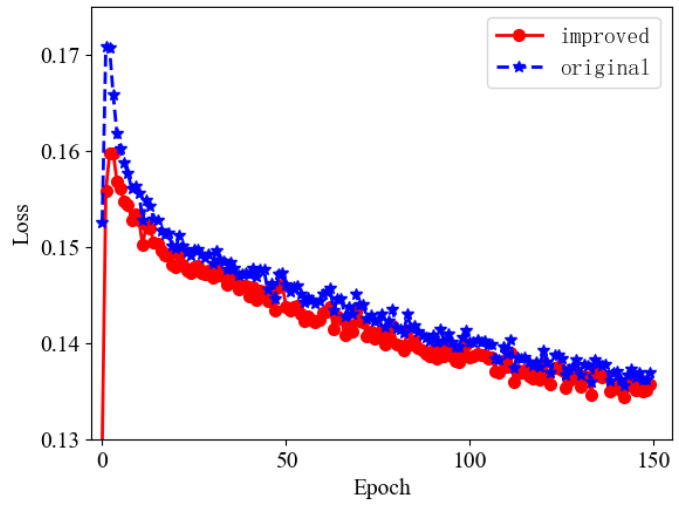
Comparison chart of the loss functions.

In order to confirm the effectiveness of the proposed improved loss function, we conducted loss function comparison experiments. During the experimental training process, we found that the curve of the loss function was stable when the epoch reached 150, so we terminated the training, and the training results are shown in Figure 11.

From the above training comparison chart, it is clear that as the number of iterations gradually increases, the curves of the loss function gradually converge, and the loss value becomes smaller and smaller. When the epoch reached 150, the loss value was basically stable. Compared with the original YOLOv5s, the regression is faster and more accurate, indicating the effectiveness of the improved algorithm.

To further verify the impact of the improved loss function on the performance of the algorithm, the YOLOv5s loss function is replaced with Focal-EIOU Loss for comparison experiments. The comparison results are shown in Table 3. From the comparison results, it can be seen that the use of Focal-EIOU Loss shows better performance, although the improvement of the mAP@0.50 value is not obvious, the mAP@0.50:0.95 is increased by 0.5%, and the FPS is increased by 13.3, proving that the Focal-EIOU Loss proposed in this paper is effective for algorithm improvement.

#### 4.3.3. Ablation Experiment

In order to further verify the effectiveness of the proposed structures, ablation experiments were conducted on the Rope3D dataset. The results of the ablation experiments are shown in Table 4. Experiment 1 is the original YOLOv5s, with a mAP@0.50 of 53.6 and an FPS of 71.4. Experiment 2 reconstructs the feature fusion layer based on the original YOLOv5s. mAP@0.50 increased by 3.5%, indicating that feature fusion layer reconstruction enhances the network feature space receptive fields, which effectively improves the detection accuracy of small targets in roadside images. At the same time, FPS increased by 14.8 and weight decreased by 71.2%; it is clear that the network structure has significant improvement in the lightweight model. Experiment 3 replaces the original pyramid with GFPN based on the former experiment. Although the improved feature fusion structure brings an increase in computation, it enhances the object detection accuracy of the algorithm. mAP@0.50 increased by 1.2%. Experiment 4 integrates the CA module into the Backbone network based on the former experiment, proving that embedding the location information into the channel attention can improve the detection performance of the model. Experiment 5 introduces Focal-EIOU Loss in the prediction head based on the former experiment. Although the mAP@0.50 only increased by 0.3%, its corresponding precision is higher than the other models, indicating that the improved loss function can help the network to improve the speed of bounding box regression and anchor box positioning accuracy.

**Figure 12:**
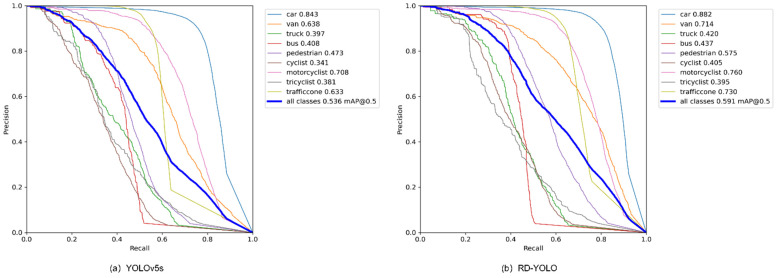
PR-Cure comparison of YOLOv5s and RD-YOLO.

**Figure 13:**
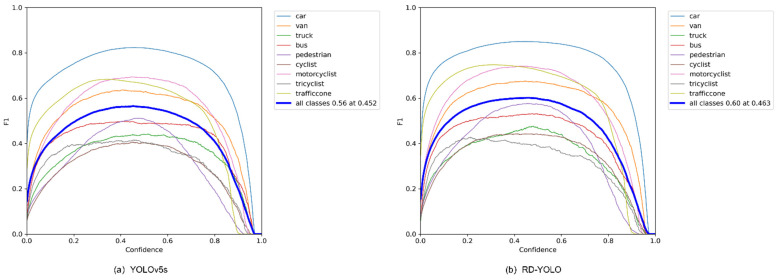
F1-Cure comparison of YOLOv5s and RD-YOLO.

Finally, we combined the above four improved structures to achieve the best performance of the detection model, and named the model RD-YOLO. Compared with the original YOLOv5s model, the mAP@0.50 value improved by 5.5% and the model weight decreased by 55.9%, while the detection speed was almost unchanged. This shows that RD-YOLO has a better detection performance than YOLOv5s and still meets the real-time requirements of industrial inspection. More actual test results are shown in Figure 12 and Figure 13.

**Figure 14:**
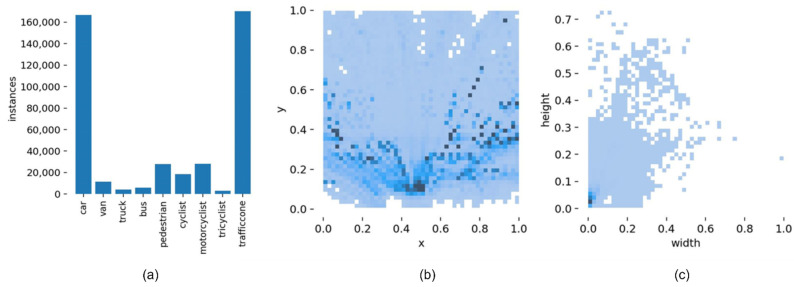
The visualization result of the analysis of the Rope3D dataset. (**a**) The distribution of detection object category and label. (**b**) The distribution of detection object center locations. (**c**) The distribution of detection object sizes.

For the analysis of the above experimental results, compared with the original YOLOv5, the proposed algorithm effectively improves the detection accuracy of each category on the Rope3D dataset. Although the improvement of the proposed algorithm for roadside object detection is obvious on the Rope3D dataset, there is still some distance between the mean average precision and ideal value. To further evaluate the dataset and analyze the reasons why the algorithm cannot achieve higher accuracy, this paper describes the visualization result of the Rope3D dataset, as shown in Figure 14. It can be seen that this dataset belongs to a difficult fine-grained classification task, the categories of pedestrian and cyclist, motorcyclist, and tricyclist have similar features, and correct detection requires a high requirement for the model. Moreover, this dataset contains a large number of small-scale target detections, especially in the case of dense and overlapping targets, which makes it difficult for the detection model to accurately identify objects. Finally, the number of samples in this dataset is relatively small for some categories, such as truck, bus, and tricycle drivers, so the object detection network does not sufficiently learn the feature information of these samples, resulting in low sensitivity and poor recognition accuracy for these samples.

#### 4.3.4. Analysis of Detection Results

In order to better demonstrate the detection effect of the improved algorithm, the improved algorithm and the current main object detection algorithms were compared and tested on the Rope3D dataset, and the experimental results are shown in Table 5. 

As can be seen from Table 2, the improved RD-YOLO algorithm in this paper has a significant improvement in mAP, model weight, and detection speed compared to the two-stage object detection algorithm Faster R-CNN. Compared with the one-stage object detection algorithms SSD, YOLOv5s, YOLOv5m, and YOLO-Z, the mAP values are improved by 34.2%, 5.5%, 4.4%, and 1.5%, respectively. The Transformer-based improved YOLOv5 model TPH-YOLOv5 effectively improves the average detection accuracy value of small targets on the VisDrone2021 dataset. To verify its effectiveness, this paper conducts experiments on the Rope3D dataset, and the results show that the AP values of each category are improved, but the detection speed is significantly slower. The model in this paper improves the mAP by 1.2% compared with TPH-YOLOv5. Moreover, it can be seen that the model in this paper has a higher detection speed, while the model weight is significantly compressed.

The algorithm proposed in this paper is compared with the current main algorithm and the latest improved algorithm. It can be found that the detection accuracy values of pedestrians, cyclists, traffic cones, and other small-scale targets are improved compared to TPH-YOLOv5. Compared with SSD, YOLOv5m, and other algorithms with more complex network models, the algorithm in this paper uses the lightweight YOLOv5s as the base model for improvement. The accuracy values of the detection results of small targets such as pedestrians, cyclists, and traffic cones still have better performance, and the detection speed is also better than these algorithms.

To make the algorithm more generalizable, comparison tests are performed on the UA-DETRAC dataset. From Table 6, it can be seen that the accuracy values of each category are improved compared to the original YOLOv5s. Although the algorithm in this paper does not guarantee the highest accuracy value for each category compared with other algorithms, it does not affect the overall average accuracy higher than other algorithms. RD-YOLO achieves higher accuracy and detection speed, while effectively compressing the model volume and improving the efficiency of model deployment in edge devices.

#### 4.3.5. Visualization of Detection Results

**Figure 15:**
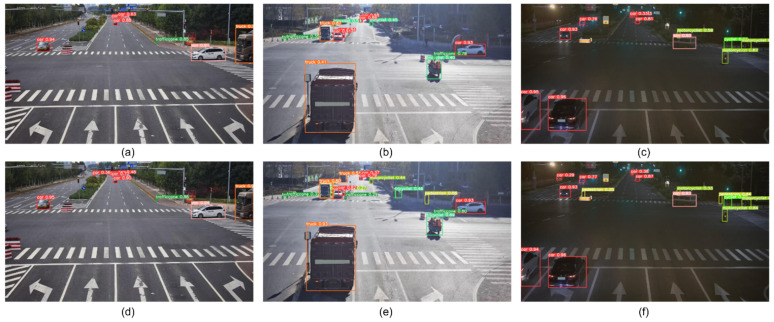
Visual comparison of YOLOv5s and RD-YOLO detection results on the Rop3D dataset. (**a**) YOLOv5s detection result of No. 1 image. (**b**) YOLOv5s detection result of No. 2 image. (**c**) YOLOv5s detection result of No. 3 image. (**d**) RD-YOLO detection result of No. 1 image. (**e**) RD-YOLO detection result of No. 2 image. (**f**) RD-YOLO detection result of No. 3 image.

In order to further verify the validity of the proposed improved algorithm, the trained model is followed to validate the Rope3D test set to obtain the visualization results, as shown in Figure 15. The upper figure shows the detection results of YOLOv5s, and the figure below shows the detection results of RD-YOLO.

As shown in Figure 15, the YOLOv5s algorithm tends to miss the detection of small targets due to the lack of shallow feature extraction. For example, in Figure 15a,d, YOLOv5s cannot detect the small-target car in the distance, but the small target is clearly detected in Figure 15d. Moreover, in occluded and low-light scenarios, the missed detection and false detection of YOLOv5s are more obvious. It can be observed from Figure 15b,e that the YOLOv5s cannot detect the targets that are not easily identified in the shadows, but the improved algorithm not only detects them accurately but also classifies them correctly. Figure 15c,f shows the target detection in a low-light road situation, where the improved algorithm better detects and identifies small-scale targets at a distance. Experiments show that the detection box obtained by the proposed algorithm in this paper fits more closely with the target to be detected, and some small targets that are not easily identified can be detected. Compared with the original YOLOv5s, the RD-YOLO reduces the missed detection rate of small-scale targets to a certain extent and improves the detection accuracy of roadside objects.

**Figure 16:**
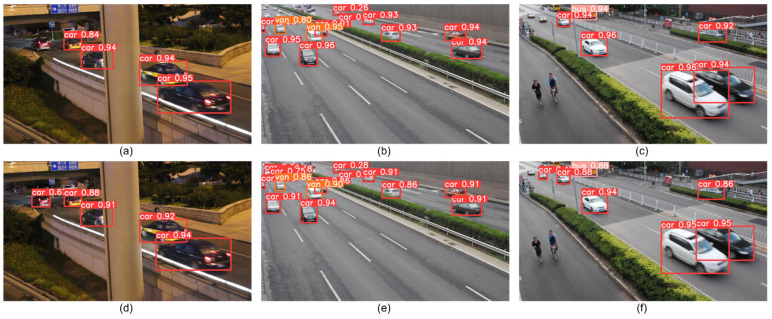
Comparison of YOLOv5s and RD-YOLO detection results on the UA-DETRAC dataset. (**a**) YOLOv5s detection result of No. 1 image. (**b**) YOLOv5s detection result of No. 2 image. (**c**) YOLOv5s detection result of No. 3 image. (**d**) RD-YOLO detection result of No. 1 image. (**e**) RD-YOLO detection result of No. 2 image. (**f**) RD-YOLO detection result of No. 3 image.

In order to make the proposed algorithm have better generalization, the YOLOv5s and RD-YOLO were validated on the UA-DETRAC test set, respectively. As shown in Figure 16, it can be seen that the improved model is significantly more accurate than the original algorithm, more sensitive to small targets, and has a lower missed detection rate whether in a dense target environment or in a complex environment such as low light at night.

To summarize, the RD-YOLO model outperforms the YOLOv5s model in detecting small-scale and dense targets and having strong robustness, resulting in superior performance and more precise detection and recognition.

## 5. Conclusions

This paper proposes an effective and efficient algorithm for roadside object detection based on YOLOv5s, which mainly solves the problem that the speed and accuracy of roadside target detection cannot be improved at the same time due to small targets, complex background, and limited feature extraction. The feature fusion layer reconstruction is proposed to more effectively capture small-target features and to achieve the lightweight design of the roadside target detection model. Then, using the GFPN for multiscale feature fusion improves the adaptability of networks to different scale features. In addition, the CA module is introduced into the Backbone network to improve the detection ability of dense small targets. Finally, the loss function is optimized to improve the speed of the bounding box regression and the positioning accuracy of the anchor box. Compared to the YOLOv5s, the RD-YOLO improves the mean average precision by 5.5% on the Rope3D dataset and 2.9% on the UA-DETRAC dataset. Furthermore, the weight of the model is reduced by 55.9% while the inference speed is almost unchanged. The algorithm proposed in this paper effectively improves the accuracy of object detection in roadside images and achieves the requirements of efficient deployment and real-time detection for roadside edge devices. 

Compared with the ideal detection requirements, our network is prone to some ambiguous targets leading to a decrease in the detection accuracy of the model. In the future, we will further optimize detection by increasing the diversity and richness of the dataset. In addition, we will continue to tune hyperparameters and optimize the model to further improve the speed and accuracy of roadside object detection.

## Figures and Tables

**Table 1:** Rope3D dataset partition table.

Dataset	Training Set	Validation Set	Total
Cloudy	10,000	3000	13,000
Night	10,000	2000	12,000
Sunny	10,000	5000	15,000
Rainy	10,000	2000	12,000

**Table 2:** Complexity and performance comparison of object detection algorithms.

Model	Params (10^6^)	GFLOPs	Weight (MB)	mAP@0.50 (%)	mAP@0.50:0.95 (%)	FPS
YOLOv5s	7.09	16.4	13.83	53.6	35.1	71.4
YOLOv5s-T	1.76	13.7	3.99	57.1	36.8	86.2
YOLOv5-G	11.1	41.0	21.5	56.6	37.1	46.2
YOLOv5-CA	7.11	16.6	13.94	54.1	35.9	70.1

**Table 3:** Performance comparison of the original and improved algorithms.

Loss	mAP@0.50 (%)	mAP@0.50:0.95 (%)	FPS
CIOU Loss	53.6	35.1	71.4
Focal-EIOU Loss	53.7	35.6	84.7

**Table 4:** Comparison of ablation experiment results.

No	Feature Fusion Layer Reconstruction	GFPN	CA	Focal-EIOU Loss	mAP@0.50 (%)	FPS	Weight (MB)
1					53.6	71.4	13.8
2	√				57.1	86.2	3.98
3	√	√			58.3	71.4	6.04
4	√	√	√		58.8	65.8	6.08
5	√	√	√	√	59.1	71.9	6.08

**Table 5:** Performance comparison of different algorithms on the Rope3D dataset.

Model	FPS	mAP (%)	Weight (MB)
SSD [14]	47.6	24.9	94.6
Faster-RCNN [12]	9.1	35.4	108.1
YOLOv5s	71.4	53.6	13.8
YOLOv5m	51.5	54.7	40.6
YOLO-Z [20]	62.7	57.6	15.2
TPH-YOLOv5 [39]	35.7	57.5	70.1
RD-YOLO	71.9	59.1	6.08

**Table 6:** Performance comparison of different algorithms on the UA-DETRAC dataset.

Model	FPS	mAP (%)	Weight (MB)
SSD [14]	56.5	57.8	92.1
Faster-RCNN [12]	6.9	65.6	108.3
YOLOv5s	53.6	94.2	14.5
YOLOv5m	35.7	95.6	42.6
RD-YOLO	61.3	97.1	6.3
